# Bio-Inspired Teleoperation Control: Unified Rapid Tracking, Compliant and Safe Interaction

**DOI:** 10.3390/biomimetics10090625

**Published:** 2025-09-16

**Authors:** Chuang Cheng, Haoran Xiao, Wei Dai, Yantong Wei, Yanjie Chen, Hui Zhang, Huimin Lu

**Affiliations:** The College of Intelligence Science and Technology, National University of Defense Technology, Changsha 410073, China; chengchuang@nudt.edu.cn (C.C.);

**Keywords:** robotic teleoperation, compliant-safe interaction, collision reaction control

## Abstract

In robotic teleoperation, the simultaneous realization of rapid tracking, compliance, and safe interaction presents a fundamental control challenge. This challenge stems from a critical trade-off: high-stiffness controllers achieve rapid tracking but compromise safety during physical interactions, whereas low-stiffness impedance controllers ensure compliant and safe interactions at the expense of responsiveness. To address this conflict, this study proposes a bio-inspired teleoperation control method (BITC) that integrates human withdrawal reflex mechanisms and the nonlinear stiffness characteristics of shear-thickening fluids. BITC features a dynamic force-feedback-driven collision reflex strategy, enabling rapid detection and disengagement from unintended contacts. Additionally, a nonlinear compliance control module is proposed to achieve both force fidelity during initial contact and adaptive stiffness modulation during progressively deeper contact in an emergency. By integrating full-state feedback tracking, the BITC teleoperation control framework is implemented to unify the performance of rapid tracking, compliance, and safety. Three experiments are conducted to demonstrate that the BITC method achieves accurate tracking performance, ensures compliant behavior during deep contact while maintaining force fidelity during initial contact, and enables safe reflexion for collision, respectively. The method is also validated to reduce peak contact forces by approximately 60% and minimizes contact duration to less than 120 ms, presenting comprehensive teleoperation performance.

## 1. Introduction

In hazardous or inaccessible environments, teleoperation systems play a critical role in domains such as disaster response [1,2], medical surgery [3], and space exploration [4,5]. Their primary function is to either replace direct human involvement or amplify operational effectiveness through remote control. In these applications, simultaneously achieving accurate trajectory tracking, compliant interaction with dynamic environments, and safe responses to sudden collisions remains a significant challenge.

**Figure 1:**
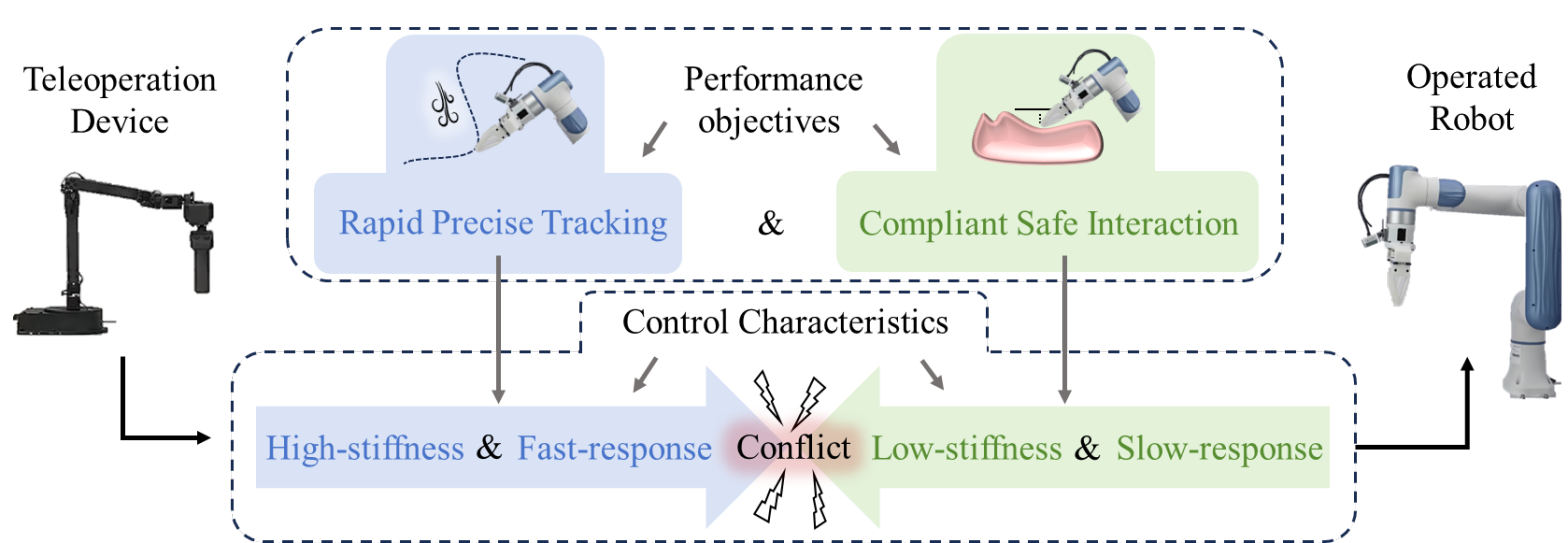
The challenges of conflicting controller characteristics driven by performance requirements in robotic teleoperation.

This necessitates a holistic control framework with dual performance requirements, as shown in Figure 1: (1) When the robot is in non-contact states, it aims to exhibit high-stiffness and fast response characteristics to ensure accurate tracking of controller commands. (2) When interacting with the environment, the system aims to switch to low-stiffness and compliant control modes to ensure interaction safety. However, traditional control strategies often prioritize one aspect at the expense of others. For example, position-based controllers (e.g., PIDs) ensure precise tracking but lack adaptability during physical interactions, potentially leading to damage in unstructured environments. Conversely, impedance control [6] and admittance control [7] emphasize compliance but may compromise tracking accuracy, especially under high-speed or variable-load conditions. The inherent contradiction between these control properties (e.g., high vs. low stiffness, fast response vs. compliance) renders a unified control strategy capable of simultaneously achieving dynamic tracking and safe interaction highly challenging. Addressing this bottleneck is essential for advancing teleoperation robots in complex real-world applications. Recent advancements in adaptive control [8] and hybrid force-position control [9] have improved robustness in uncertain environments. However, these methods often fail to address emergency collision scenarios, where rapid, reflex-like responses [10] (e.g., human-like “retraction”) are essential to prevent mechanical damage or environmental hazards [11]. For instance, in high-speed teleoperation tasks (e.g., demolition or assembly), a sudden collision with an unexpected obstacle can cause excessive force spikes [12], risking system instability or safety breaches [13].

In the operational tasks considered in the above analysis, the conflicting performance objectives, rapid tracking, compliance, and safe interaction, pose a significant challenge for normalized controllers. Specifically, these controllers must rapidly adapt their control characteristics to accommodate varying interaction scenarios, often requiring substantial adjustments within a short time frame. To address this issue, we draw inspiration from two biological phenomena: the human withdrawal reflex and the properties of shear-thinning fluids (STF-n) [14]. The human withdrawal reflex is a spinal reflex that enables a rapid, involuntary withdrawal of the limb upon encountering a harmful stimulus, effectively preventing prolonged injury. STF-n is the type of non-Newtonian fluid whose viscosity decreases with an increase in the shear rate (or shear stress); common examples include ketchup and blood [15]. We observed that when the manipulator is subjected to a collision, its stiffness and damping (similar to the shear rate in shear-thinning fluids (STF-n), which increases under stress) are reduced, enabling rapid reverse responses. This objective strategy aligns with the fluid-like compliance observed in STF-n under high shear stress. Therefore, this paper proposes a bio-inspired teleoperation control (BITC) method based on a combination of the state feedback and compliant interaction for teleoperated manipulators. The method integrates three key components:

(1) Rapid Trajectory Tracking: ensures the synchronization of master-slave tracking across varying velocities, while reducing overshoot under unpredictable motions.
(2) Compliant Interaction Control: dynamically adjusts stiffness and damping based on real-time force feedback, enabling smooth and safe contact with objects.
(3) Collision Reflex Mechanism: mimics human reflexes by triggering an emergency retraction when abrupt force changes (e.g., collisions) are detected, preventing excessive interaction forces.

The proposed control method is validated through experiments involving variable-velocity tracking, slow contact, and high-speed collision scenarios. Quantitative results demonstrate its superiority over existing common methods in balancing tracking accuracy, compliance, and safety, particularly in dynamic and unpredictable environments.

The structure of the subsequent sections is as follows: In Section 2, we present a review of related work, focusing on existing research in adaptive control for various interaction scenarios. Section 3 introduces the proposed BITC method in detail. Section 4 describes the three different experimental setups designed to evaluate rapid tracking, interaction compliance, and collision safety, along with the corresponding results analysis. Finally, Section 6 provides the conclusions of this paper.

## 2. Related Work

Since the inception of teleoperation systems, tracking accuracy, interaction compliance, and operational safety have remained central focuses of research and applications. Numerous scholars have conducted ongoing studies in these areas. The related research is systematically reviewed based on two key aspects: (1) adaptive tracking and compliance control; (2) collision safety with human-like reflex mechanisms.

### 2.1. Adaptive Tracking and Compliance Control

Benefiting from the integration of servo control systems and impedance/admittance control frameworks, research on tracking accuracy and operational compliance within single-task scenarios has reached maturity [16,17]. However, as previously highlighted, the inherent incompatibility between high-stiffness fast-response dynamics and low-stiffness slow-response characteristics imposes fundamental limitations on their performance in complex interaction tasks. To enhance robustness in uncertain environments, adaptive variable control has been widely adopted. For example, a hybrid force/position control method investigated in [18] for a bimanual teleoperation system enables a motion synchronization effect in a soft-handling environment. The self-tuning impedance controller developed in [8,19] adjusts stiffness and damping based on environmental feedback to balance accuracy and compliance. Furthermore, the variable impedance characteristics of the human arm received attention [20]. Humans exploit their spring-like muscle characteristics to regulate their end-point arm force and impedance continuously [21]. This explains the unique ability that humans possess to interact skillfully even with unstable environments that involve tool use or divergent force fields [22], which inspired the development of continuous variable impedance control, and has achieved an adaptive trade-off between precision and compliance manipulation [23]. Moreover, variable impedance control based on passivity theory [24], robust control [25], and shared learning-based control [26] has emerged to extend compliance and enhance stability and operational capabilities in complex interaction scenarios.

While the aforementioned adaptive variable control methods indeed enable spatial and temporal compliance and flexibility, no existing research has addressed both compliant interaction and collision safety within a unified framework.

### 2.2. Safety Mechanism Under Collision Situation

Implementing collision safety is critical for teleoperation systems and could significantly reduce operator cognitive load [12]. Recent work by Michel et al. [27] proposed a hybrid control scheme integrating adaptive impedance control with collision-triggered switching, which demonstrated enhanced capability in detecting and compensating for abrupt collision events. The collision reflex requires rapid motion and high dynamic responsiveness, yet it is prone to secondary collision risks due to excessive force generation or directional misalignment, while also posing challenges to system stability. A controller enabling end-effector motion after collision is proposed [28], which leverages a dynamically consistent null space projector along a specific Cartesian direction while minimizing disturbances in other directions. Meanwhile, the collision-triggered framework was further extended to bilateral teleoperation using a tank-based deployment, where system stability was rigorously validated [29]. Vorndamme et al. systematically evaluated diverse reflex strategies (e.g., admittance, retraction) through a multi-dimensional “Reflex Space” framework, quantifying trade-offs in performance metrics like peak force, contact time, and post-reflex speed [30]. Building on this foundation, Vorndamme et al. formalized these insights into the Robot Safety Assessment Pipeline (RSAP), which provided a standardized methodology for reflex design and certification. By defining measurable safety variables and context-specific reflex elements, RSAP bridges empirical evaluations with systematic safety validation, enabling objective comparisons across reflex strategies [10]. While significant progress has been made in collision reflex response strategies, existing approaches predominantly focus on immediate reaction mechanisms during impact events, with relatively less attention paid to the recovery phase and the resumption of continuous operations post-collision. Notably, references [10,30] provided a comprehensive categorization and evaluation of established methods. However, their comparative analyses primarily focus on well-established control paradigms. A unified theoretical framework that integrates tracking compliance transitions with collision response dynamics remains an open challenge, highlighting a critical avenue for future research.

Biologically inspired control strategies have gained increasing attention for their ability to mimic human reflexes. Among these, muscle-impedance models [31] have demonstrated the ability to adjust compliance dynamically by integrating physiological principles. These models have proven particularly useful in precision- and safety-critical applications like surgical robotics. However, their application to high-speed teleoperation remains limited due to computational complexity. Regarding the rapid change in contact physical properties over short periods (collision times are typically very short), non-Newtonian fluids represent a paradigm in material science that provides inspiration. Existing work, such as that by Chen et al. [32], has applied this principle to human-robot interaction (HRI) by leveraging the properties of shear-thickening fluids (STF-c), where stiffness and damping increase exponentially with velocity. This approach maintains rigidity and damping during high-speed external disturbances to reject perturbations while preserving low-speed traction for smooth interaction with the target. However, we observed that the requirement for collision-induced compliance operates in the opposite manner: during collisions, stiffness and damping must be reduced to enable rapid reverse reactions, aligning with the characteristics of shear-thinning fluids (STF-n).

Therefore, the BITC control method proposed in this paper aims to emulate human reaction mechanisms (similar to the human reflex arc) by introducing a collision reflex module that integrates real-time force monitoring with rapid trajectory correction. Unlike traditional event-triggered approaches, it employs a soft threshold mechanism to detect sudden force changes, triggering a contraction within milliseconds. Additionally, inspired by the properties of shear-thinning fluids, this work incorporates nonlinear stiffness-damping adjustments to achieve faster collision responses without compromising stability.

## 3. Method

**Figure 2:**
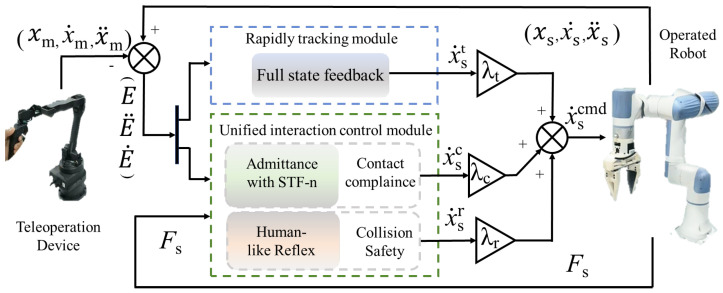
The overview of the proposed BITC control framework.

To achieve manipulation control that simultaneously realizes a fast and precise tracking performance as well as collision safety and a compliant contact interaction performance, bio-inspired teleoperation control (BITC) is proposed. The overview of BITC is shown in Figure 2. The control command $x_{s}^{\mathrm{cmd}}$ in the teleoperation system for the slave manipulator mainly consists of three parts:

(1) $$x_{s}^{\mathrm{cmd}}=\lambda_{t}{\dot{x}}_{s}^{t}+\left(\lambda_{c}{\dot{x}}_{s}^{c}+\lambda_{r}{\dot{x}}_{s}^{r}\right),$$

where ${\dot{x}}_{s}^{t}$ represents the tracking control component for the non-contact movement scenario, ${\dot{x}}_{s}^{c}$ denotes the compliance control component for the sustained contact situation, and ${\dot{x}}_{s}^{r}$ denotes the reaction control component for the collision safety requirement. $\lambda_{t}$ serves as the weighting factor for the tracking component within the entire controller architecture, enabling integrated tuning of performance trade-offs between tracking accuracy, interaction fidelity, contact compliance, and collision safety.

The rapid tracking control module is primarily implemented through full-state feedback to address the challenge of tracking the trajectory of the slave manipulator relative to the master controller’s manipulated path. It requires rapid response and precise control, while also avoiding overshoot caused by motion discontinuities or uncertainties.

The unified adaptive interaction control module is composed of impedance with STF-n for contact compliance and human-like reflexes for collision safety to address three key characteristics during the interaction between the slave device and the environment:

- Fidelity: When the slave device makes initial contact with the environment or an object, it is important to maintain accurate force feedback to ensure the high fidelity of the interaction.
- Compliance: During prolonged contact, following the servo tracking command may lead to a rapid increase in interaction forces, which poses a risk of damaging the equipment. In such cases, compliant control must be introduced to ensure safe and sustained interaction.
- Safety: In the event of operator error or unexpected external collisions, a rapid stress-response mechanism is required to minimize impact forces and ensure the safety of both the slave device and the interacting objects.

### 3.1. Rapid Tracking Control

In teleoperation systems, velocity tracking control and impedance control are commonly used master-slave teleoperation control strategies. As analyzed in the Section 2, velocity tracking exhibits characteristics of high stiffness and low compliance, while impedance control shows the opposite traits: high compliance and low stiffness. To maintain motion accuracy, the velocity tracking strategy is applied. However, unlike conventional open-loop velocity tracking or position-closed-loop velocity tracking approaches, we introduce state-closed-loop alignment. The velocity tracking control of the slave is primarily targeted at the state velocity of the master, and the tracking velocity is corrected through closed-loop adjustments at three levels: acceleration, velocity, and the position between the master and slave systems. The control variable of the tracking part ${\dot{x}}_{s}^{t}$ is designed as follows:

(2) $${\dot{x}}_{s}^{t}={\dot{x}}_{m}+k_{p}^{t}E+k_{v}^{t}\dot{E}+k_{a}^{t}\ddot{E}$$

where $E=x_{m}-x_{s}$,  $\dot{E}={\dot{x}}_{m}-{\dot{x}}_{s}$, $\ddot{E}={\ddot{x}}_{m}-{\ddot{x}}_{s}$ represents the position, velocity, and acceleration errors between the master and slave devices. $(x_{m},{\dot{x}}_{m}$ and ${\ddot{x}}_{m}),\left(x_{s},{\dot{x}}_{s},{\ddot{x}}_{s}\right)$ denote the movement state of the teleoperation device and operated robot, respectively. Among the three states, position and velocity are obtained through encoder readings, while acceleration is calculated by differentiating the velocity data. To reduce noise, the acceleration signal requires low-pass and median filtering. $k_{p}^{t},k_{v}^{t},k_{a}^{t}$ denote the control gains for three types of errors, respectively. They can be tuned sequentially: first $k_{p}^{t}$, then $k_{v}^{t}$, and finally $k_{a}^{t}$. Specifically, $k_{p}^{t}$ primarily influences tracking accuracy and steady-state error but may cause overshoot; $k_{v}^{t}$ could improve the response speed, but may introduce oscillations; and $k_{a}^{t}$ helps reduce the impact caused by communication delays, but the gain should not be too large to avoid divergence.

### 3.2. Unified Interaction Control: Contact Compliance and Collision Safety

To meet the requirements of normal interaction fidelity, continuous interaction compliance, and collision safety, the unified interaction control based on the biological human-like reflex (hand-withdraw) mechanism in collision emergency and the properties of shear-thinning fluids  (STF-n) is proposed.

(3) $${\dot{x}}_{s}^{c}=\underset{\mathrm{Contact compliance}}{\underset{︸}{\lambda_{c}\left(k_{f}^{t}{\left(F_{s}/F_{\mathrm{th}}\right)}^{l}-D{\dot{x}}_{s}^{n}-KE^{m}\right)}}\;+\underset{\mathrm{Collision safety}}{\underset{︸}{\lambda_{r}{\hat{\dot{F}}}_{s}e^{-k_{e}t}\mathrm{flag}\left({\hat{\dot{F}}}_{s}\right)}}\;$$

where $\lambda_{c}\left(k_{f}^{t}{\left(F_{s}/F_{\mathrm{th}}\right)}^{l}-D{\dot{x}}_{s}^{n}-KE^{m}\right)$ denotes the **contact compliance** control component during sustained interaction, with $k_{f}^{t}$ as the compliant force gain coefficient.

**Figure 3:**
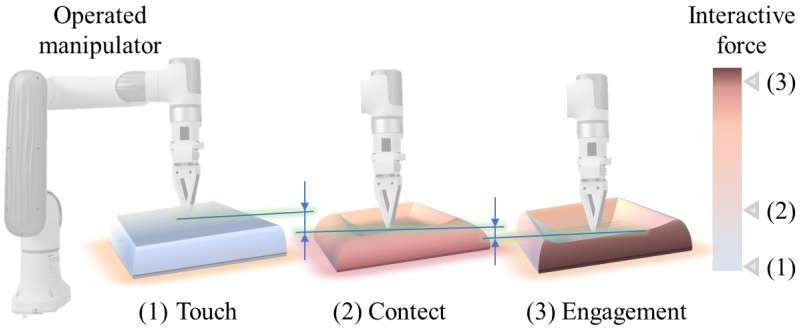
Schematic illustration of the interaction process between a robot and an object during slow contact: the contact force increases nonlinearly and rapidly as the contact depth increases.

This design draws inspiration from STF-n: when contact forces are small, this control component maintains a low gain to minimize interference with trajectory tracking, ensuring that the slave manipulator interacts with the environment in alignment with human intent and preserving contact force fidelity. As the slave manipulator continuously tracks the motion of the master manipulator, the contact depth progressively increases, causing the interaction force to transition from the touch phase (also known as the initial contact phase) to the contact phase (as illustrated in Figure 3). Since the interaction force exhibits a nonlinear dependence on contact depth, the interaction force $F_{s}$ poses a surge risk when the value is up to the threshold $F_{\mathrm{th}}$. After this critical point, the control term ${\left(F_{s}/F_{\mathrm{th}}\right)}^{l}$ compliance-inducing torque. The quadratic amplification of this term enhances the compliance effect, rapidly shifting the system from precise tracking and contact fidelity modes to a compliant interaction phase, thereby preventing unbounded force growth. To ensure stability in the compliance-inducing torque, nonlinear damping and stiffness terms $-D{\dot{x}}_{s}^{n}-KE^{m}$ are based on the smooth access characteristics of STF-n, which are particularly suitable for compliance control in scenarios with large sustained contact forces. By setting the exponents *n* and *m* within the range 0<n<1 and 0<m<1, the damping-stiffness term $-D{\dot{x}}_{s}^{n}-KE^{m}$ exhibits a behavior that is initially dominant and gradually attenuates over time. In the initial phase, this term effectively preserves contact force fidelity, ensuring accurate interaction with the environment. As the interaction progresses, when compliance control is required to ensure contact safety, the system’s stiffness and damping terms are suppressed by *n* and *m*, facilitating the effectiveness of the compliance-inducing torque to transition the system into a compliant regime. Subsequently, after completing the compliant regime transition, the stiffness-damping components reassert their role through energy dissipation mechanisms, ultimately driving the system toward convergence.

The term $\lambda_{r}{\hat{\dot{F}}}_{s}e^{-k_{e}t}\mathrm{flag}\left({\hat{\dot{F}}}_{s}\right)$ represents the **collision safety** control component, where $\mathrm{flag}\left({\hat{\dot{F}}}_{s}\right)$ denotes the collision detection function. The collision reflex module promptly generates a reverse velocity control command upon detecting a collision, enabling the operated manipulator to rapidly disengage from the contact surface. This control command persists and decays over time, mimicking the human withdrawal reflex in its motion pattern. The generation process of the collision reflex control term is illustrated in Algorithm 1. The process begins by inputting the operated manipulator’s current end-effector contact force and timestamp, followed by computing the force derivative using a finite difference method (denoted as ${\hat{\dot{F}}}_{s}$). A collision is detected if two conditions are simultaneously satisfied: (1) the absolute value of the force derivative ${\hat{\dot{F}}}_{s}$ exceeds a predefined threshold $C_{\mathrm{th}}$, indicating a sudden change in contact force, and (2) the current force magnitude is greater than its previous value, ensuring that force reductions during retraction (e.g., due to environmental compliance) do not trigger false positives. When these conditions are met, the flag of collision flag(·) is set to 1, initiating the collision reaction mechanism. At this point, the collision reaction force configured by this control module is defined as $f_{c}$ and initialized to the current force differential ${\hat{\dot{F}}}_{s}$, and the reaction start time $t_{0}$ is recorded. For subsequent collision events within the reaction window, $f_{c}$ is updated to the new ${\hat{\dot{F}}}_{s}$ when the new value exceeds the existing $f_{c}$; otherwise, both $f_{c}$ and $t_{0}$ remain unchanged to avoid conflicts from overlapping collisions. While the collision flag remains active (flag(·)), the time interval $t=t_{c}-t_{0}$ is calculated. When *t* falls within the predefined reaction duration $T_{\mathrm{decay}}$, the collision reaction control component $\lambda_{c}{\hat{\dot{F}}}_{s}e^{-k_{e}t}\mathrm{flag}\left({\hat{\dot{F}}}_{s}\right)$ is activated. This component initially peaks at maximum magnitude and decays exponentially over time, driving the system to rapidly retract from the collision at the highest velocity, followed by a gradual deceleration to ensure smooth re-engagement with the environment. Once *t* exceeds $T_{\mathrm{decay}}$, the collision flag is reset to 0, terminating the reaction phase and resetting the system to await future collision events.
**Algorithm 1** Collision safety module**Input:**
 $F_{s}$, $t_{c}$**Output:**
 The control variables of the collision safety module ${\dot{x}}_{s}^{r}$.1:${\hat{\dot{F}}}_{s}=\frac{F_{s}\left(n\right)-F_{s}\left(n-1\right)}{T_{\mathrm{sample}}}$.2:**if** (${\hat{\dot{F}}}_{s}>C_{\mathrm{th}}$) **and** ($\mathrm{abs}\left(F_{s}\left(n\right)\right)>\mathrm{abs}\left(F_{s}\left(n-1\right)\right)$) **then**3:   $\mathrm{flag}(F_{s})=1$, there is a collision.4:   **if** (${\hat{\dot{F}}}_{s}>\mathrm{abs}\left(f_{c}\right)$) **then**5:     $f_{c}={\hat{\dot{F}}}_{s}$, $t_{0}=t_{c}$. Update the collision reaction force and the onset of action time.6:   **else**7:     $f_{c}=f_{c}$. Maintain previous collision impulse values; $f_{c}$ remains unchanged.8:   **end if**9:**else**10:   $\mathrm{flag}(F_{s})=0$, there is no collision.11:**end if**12:**if** ($\mathrm{flag}(F_{s})=1)$ **then**13:   $t=t_{c}-t_{0}$14:   **if** ($t<T_{\mathrm{decay}}$) **then**15:     ${\dot{x}}_{s}^{r}={\hat{\dot{F}}}_{s}e^{-k_{e}t}\mathrm{flag}\left(F_{s}\right)$16:   **else**17:     $\mathrm{flag}(F_{s})=0$, $f_{c}=0$, ${\dot{x}}_{s}^{r}=0$18:   **end if**19:**else**20:   ${\dot{x}}_{s}^{r}=0$21:**end if**22:**return** ${\dot{x}}_{s}^{r}$

In summary, our control law integrates multiple computational components through superposition principles. This continuous blending of tracking, compliance and reaction control terms ensures relatively smooth operation across all interaction phases, eliminating abrupt transitions while maintaining system stability within a unified framework. In the compliant-control phase for sustained deep contact, the properties of shear-thinning fluids are embedded. The corresponding control component increases nonlinearly with rising contact force or with the rate of change of the motion state. This component is superimposed on the tracking control component to yield the total control output, eliminating the need for control-law switching and ensuring a smooth transition from tracking to compliance. For rapid-collision reaction control, once a collision is detected, the derivative of the collision force serves as the input for the reaction module, enabling a large control gain at the instant of impact that drives the manipulator away from the collision source. The module determines whether its control contribution is added to the overall control law based on the collision-detection result. An introduced reaction-decay mechanism maintains a sustained reaction control magnitude after the collision signal is received, preventing gradual decay and thus avoiding secondary collisions caused by excessive reaction motions. Owing to superposition with the tracking control magnitude, the attenuation of the safety-reaction component allows the manipulator to return smoothly to its original task pose. The detailed parameter-tuning methodology is provided in Appendix A.

The control command for the slave system’s operational velocity is synthesized from the combined contributions of the rapid tracking, contact compliance, and collision reflex modules. This velocity command is ultimately converted into individual joint motor velocity commands for the robotic arm via inverse kinematics, which then drives the arm’s motion through motor velocity servo control.

## 4. Experiments and Results

### 4.1. Setup

To validate the effectiveness and performance of the proposed method in achieving rapid, accurate tracking and safe, compliant teleoperation, the three experiments, including unconstrained tracking, slow contact, and rapid collision, were designed:

(1) Unconstrained Tracking Experiment: The operator manipulates the slave manipulator via the master device (Dobot Xtrainer) to perform back-and-forth lateral movements. The tracking speed and precision of the slave manipulator’s trajectory following the master command are compared under different control methods.
(2) Slow Contact Experiment: The operator guides the slave manipulator to make gradual contact with a soft material while incrementally increasing the contact depth. This tests the following factors: Force Fidelity: the accuracy of the slave manipulator’s force feedback during initial contact. Compliance Performance: the adaptability of the slave manipulator to maintain smooth interaction as contact depth increases.
(3) Rapid Collision Experiment: The operator drives the slave manipulator to collide with an object at high speed. This evaluates the system’s safety response capability under extreme interaction scenarios across different control methods.

**Figure 4:**
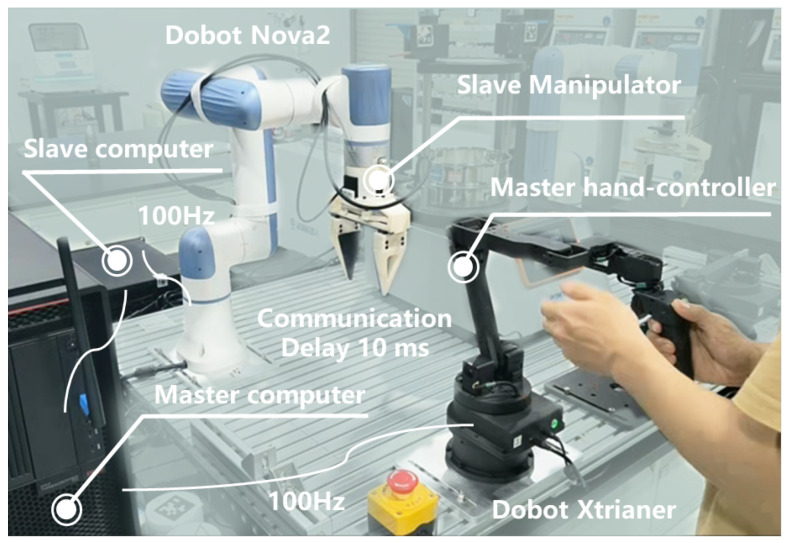
The teleoperation system for experiments.

The teleoperation system for experiments is shown as Figure 4.

The master teleoperation device is a Dobot Xtrainer haptic controller, which features a 6-DOF serial-link structure actuated by servo motors. A 6-axis force-torque sensor is integrated into the end-effector to provide real-time interaction feedback. A 1:2 position mapping ratio is implemented between the master and slave devices to ensure proportional motion scaling. The system employs separate computers for master and slave control, connected via the ROS (Robot Operating System) framework. The control frequency operates at approximately 100 Hz, with an average communication delay of around 10 ms between the master and slave devices. The parameters of the BITC are set as $\left\{\lambda_{t},k_{p}^{t},k_{v}^{t},k_{a}^{t}\right\}=\left\{0.88,1.15,0.17,0.065\right\}$, $\left\{\lambda_{c},k_{f}^{t},F_{\mathrm{th}},l,m,n,D,E\right\}=\left\{0.25,1.3,2,2.2,0.5,0.7,1.6,0.21\right\}$; $\left\{\lambda_{r},C_{\mathrm{th}},k_{e}\right\}=\left\{0.043,15,1.68\right\}$. Some experimental recordings and functional demonstrations can be found in the Supplementary Material video.

### 4.2. The Unconstrained Tracking Experiment

**Figure 5:**
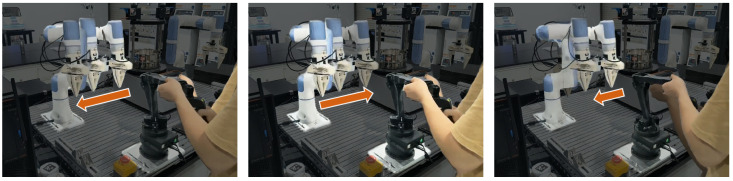
The unconstrained tracking teleoperation experiment. Red arrows are used to indicate the movement direction of the manipulator.

To validate the effectiveness of the proposed teleoperation method in achieving rapid and accurate tracking, the unconstrained tracking teleoperation experiment is conducted as shown in Figure 5. To ensure consistency in testing, the master device is programmed to perform harmonic lateral movements at four velocities (0.25 m/s, 0.5 m/s, 0.75 m/s, and 1 m/s), while the slave manipulator is tasked with replicating these motions. Five control strategies are implemented for performance comparison: (1) open-loop velocity tracking (OL-VT), (2) closed-loop velocity tracking with position feedback (PL-VT), (3) impedance control with high stiffness (Imp-H), (4) impedance control with low stiffness (Imp-L), (5) recent adaptive variable impedance control (V-Imp) [27] and (6) the proposed bio-inspired teleoperation control (BITC). The end-effector position of the slave manipulator and the master command trajectory is recorded to compute tracking errors under varying conditions.

**Figure 6:**
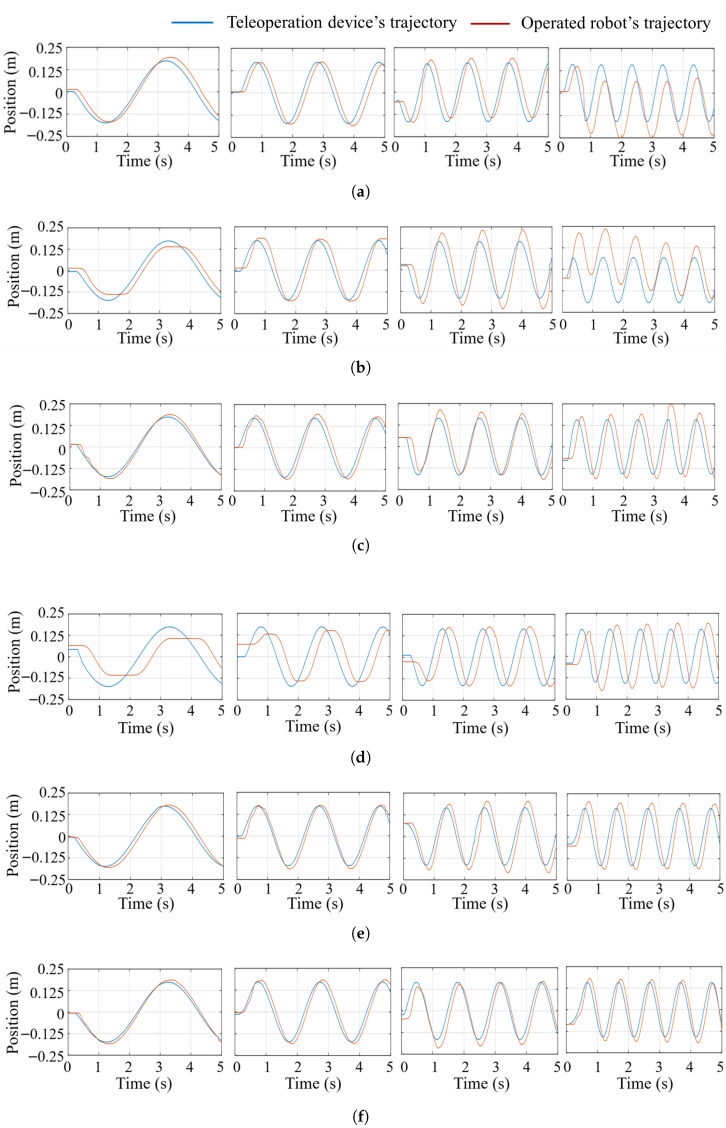
Comparison of trajectory tracking curves of the slave manipulator under harmonic lateral motion at four velocities (0.25 m/s, 0.5 m/s, 0.75 m/s, and 1 m/s) across different teleoperation methods. Each column (from left to right: Columns 1–4) corresponds to a specific velocity). (**a**) Open-loop velocity tracking (OL-VT), (**b**) Closed-loop velocity tracking with position feedback (PL-VT), (**c**) impedance control with high stiffness (Imp-H), (**d**) impedance control with low stiffness (Imp-L), (**e**) adaptive variable impedance control (V-Imp) [27], (**f**) state feedback and compliant interaction (BITC).

The tracking curves shown in Figure 6 demonstrate distinct performance characteristics across control strategies and velocities. At low speeds (0.25–0.5 m/s), the Imp-H method outperforms Imp-L due to its higher stiffness, while PL-VT achieves superior accuracy compared to OL-VT and Imp-H. Notably, the proposed method matches the performance of PL-VT under these conditions. However, as velocity increases to 0.75–1 m/s, OL-VT and Imp-L exhibit significant lag, and PL-VT begins to show overshoot at 1 m/s. In the high-speed regime, Imp-H experiences a combination of lag and overshoot, despite its stiffness being preserved. In contrast, the proposed method consistently achieves precise tracking without noticeable lag or overshoot, with tracking errors remaining below 5% across all tested velocities.

**Figure 7:**
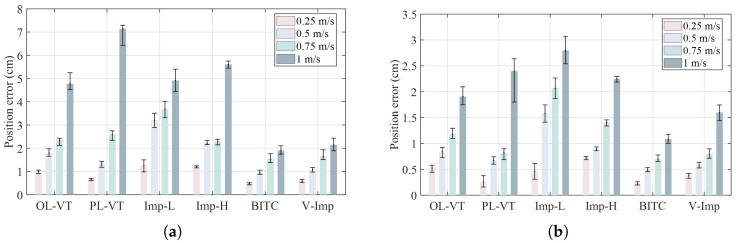
Statistical results from 10 repeated trials of each test. (**a**) The mean and error band of maximum position error from multiple trials in the tracking experiment. (**b**) The mean and error band of average position error from multiple trials in the tracking experiment.

The quantitative absolute and mean tracking error of the 24 trials are presented in Table 1. At a speed of 1 m/s (beyond the primary test range), the proposed method achieves a maximum error of 1.91 cm and an average error of 1.08 cm, representing a 40% reduction compared to the second-best method V-Imp [27] (The maximum error is 2.91 cm, the average error is 1.47 cm). These quantitative results further highlight the proposed BITC method’s robustness to high-speed operations and its ability to suppress overshoot during reciprocating motions. We conducted 10 repeated trials, plotted the statistical summaries in Figure 7, and observed that the results align with the previous analysis.

### 4.3. The Slow Contact Experiment

**Figure 8:**
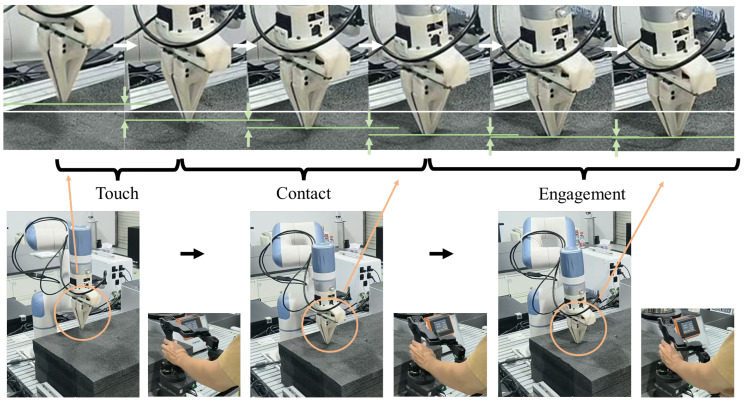
The slow contact teleoperation experiment.

To evaluate the proposed control method’s ability to achieve force fidelity during initial contact and compliant safety during deep penetration, a slow contact teleoperation experiment was conducted (see Figure 8). This experiment compared the following methods: position closed-loop tracking (PL-VT), low-stiffness impedance control (Imp-L), a recent variable impedance control method (V-Imp) [27], and the proposed BITC method. in which the slave manipulator performed vertical downward motion at low speeds, tracking the master end movement to interact with a soft black foam block. Three trials are designed to increase contact speed and depth incrementally:

- Trial 1: The end of the master device moves at an operational speed of 0.5 mm/s, with a target penetration depth of 1 cm below the contact surface.
- Trial 1: The end of the master device moves at an operational speed of 1 mm/s, with a target penetration depth of 1 cm below the contact surface.
- Trial 3: The end of the master device moves at an operational speed of 1 mm/s, with a target penetration depth of 1.5 cm below the contact surface.

Real-time interaction forces were measured using a 6-axis force-torque sensor mounted on the end-effector of the operated manipulator.

**Figure 9:**
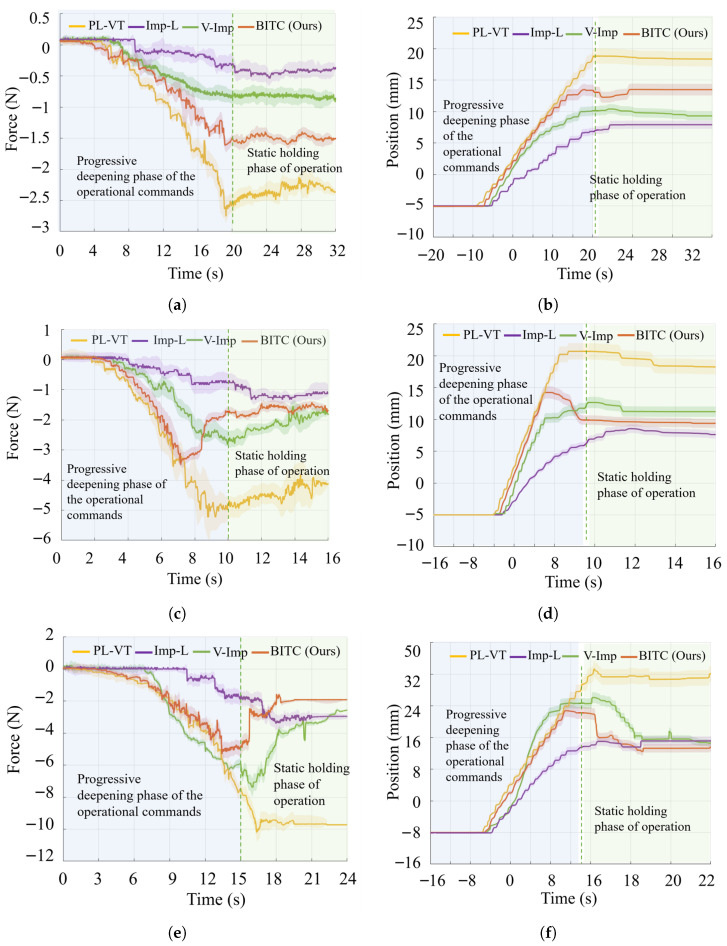
Comparison of operated manipulator interactive force curves in the slow contact experiment between various teleoperation methods with different speeds. (**a**) Contact force in Trial 1 (1 mm/s, 2 cm penetration). (**b**) End effector position in Trial 1. (**c**) Contact force in Trial 2 (2 mm/s, 2 cm penetration). (**d**) End effector position in Trial 2. (**e**) Contact force in Trial 3 (2 mm/s, 3 cm penetration). (**f**) End effector position in Trial 3.

The experimental results, as illustrated in Figure 9, highlight critical differences in force profiles across methods. The yellow curve (PL-VT) exhibits the highest interaction forces, escalating rapidly with increasing contact velocity and depth. This nonlinear force growth underscores the risks of deep penetration, particularly under master-side misoperation or lack of force feedback. The absolute values of the purple curve (Imp-L) are significantly smaller than those of the yellow curve. As the master manipulates the target deeper, the slave contact force increases relatively slowly, ensuring safer interaction. The green curve (V-Imp) method lies between the purple and red curves, demonstrating both adaptability and compliance. However, during the initial contact phase, its alignment with the yellow curve is less precise compared to the red curve. Notably, in Test 3, the adaptive nature of the V-Imp method allowed its tracking trajectory to approach the yellow curve more closely than in the other two tests, though the conformity still lags slightly behind. The red curve represents the interaction force achieved using the BITC. During initial contact, its force profile closely matches that of the yellow curve (PL-VT). As the interaction force gradually increases, the proposed method deviates from strict slave-to-master trajectory tracking, avoiding the accelerated force escalation observed in the PL-VT tracking method during late-stage contact. This demonstrates that both the proposed method and impedance control exhibit compliance. The absolute values of the purple curve are significantly smaller than those of the yellow curve. As the master manipulates the target deeper, the slave contact force increases relatively slowly, ensuring safer interaction. The red curve represents the interaction force achieved using the proposed method. During initial contact, its force profile closely matches that of the yellow curve (position-closed-loop velocity tracking method). As the interaction force gradually increases, the proposed BITC method deviates from strict slave-to-master trajectory tracking, avoiding the accelerated force escalation observed in the position-closed-loop velocity tracking method during late-stage contact. This indicates that the BITC method exhibits compliance; compared to impedance control (which also provides compliance), BITC achieves superior interaction fidelity. When the operator intentionally guides the manipulator to contact the environment, BITC ensures sufficient contact force without causing unintended deformation during interaction.

The quantitative results of the maximum dynamic contact force at the slave device during the progressive deepening phase and the average static force at the slave device during the static holding phase are presented in Table 2.

Notably, comparing the three experimental scenarios reveals that increased velocity and deeper target trajectory penetration significantly amplify both the dynamic peak contact force and steady-state contact force in the position-closed-loop velocity tracking method. Impedance control exhibits a similar trend in the contact force increase, albeit with a lesser magnitude of the contact force. However, the BITC method maintains low growth rates for both force metrics while preserving high-fidelity tracking during the initial contact phase. The quantitative results of the maximum dynamic contact force at the slave device during the progressive deepening phase and the average static force at the slave device during the static holding phase are presented in Table 2. The steady-state contact force in the BITC method remains below 2 N across three experimental conditions (Trial 1: 1 mm/s operational speed, 2 cm penetration depth; Trial 2: 2 mm/s operational speed, 2 cm penetration depth; Trial 3: 2 mm/s operational speed, 3 cm penetration depth). As the experiments progressed from Trial 1 to Trial 3, both the operational speed and penetration depth incrementally increase. However, the growth rate of the steady-state contact force in our method (from −1.558 N to −1.893 N to −1.977 N) is significantly smaller than that of the Imp−L method (from −0.474 N to −1.182 N to −3.103 N) and V-Imp method (from −0.774 N to −1.927 N to −2.652 N). Notably, even under the most demanding Trial 3 conditions, the steady-state contact force of BITC remains lower than that of the impedance control method.

Additionally, the quantitative analysis results can be derived from the comparison of the Figure 9a,c,e and Table 2. For PL-VT, dynamic peak forces surge from 2.745 N in Trial 1 to 10.26 N in Trial 3, while stable contact forces rise proportionally. Imp-L shows a moderate force increase but compromises initial tracking fidelity. The performance of V-Imp lies between the two, with dynamic peak forces surging from 0.839 N in Trial 1 to 7.590 N in Trial 3. The BITC method, however, limits dynamic forces to <5.5 N and the absolute average static contact forces at 1.5–2 N, even under higher velocities and depths. Notably, in Trials 2 and 3, stable forces remain below 2 N, outperforming Imp-L. These results confirm the BITC method’s consistent compliance, safety, and fidelity across varying contact conditions.

### 4.4. The Collision Reflex Experiment

**Figure 10:**
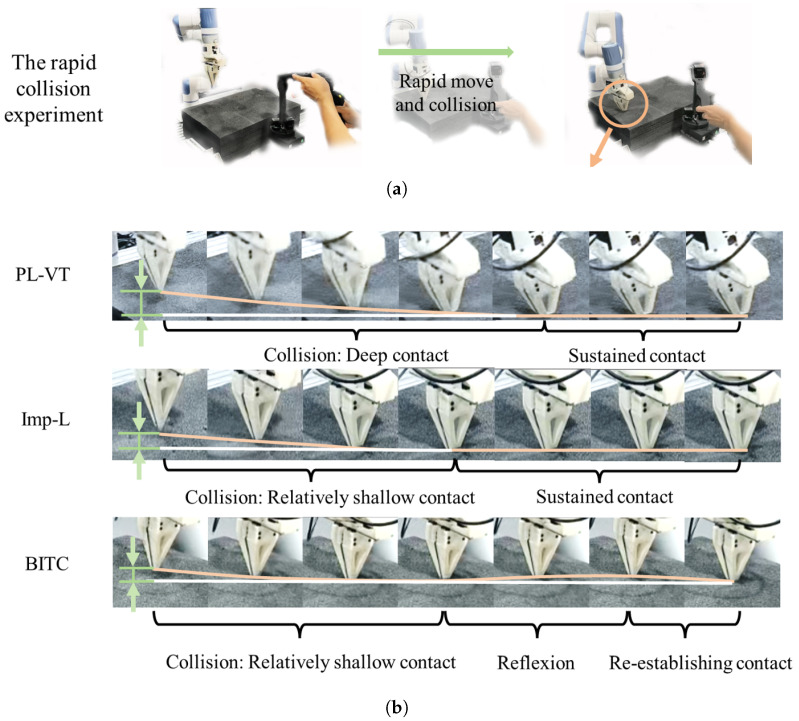
The collision reflex experiment. (**a**) Experimental procedure and setup. (**b**) Microscopic magnified perspective of collision processes using different methods.

To validate the proposed method’s collision reflexion safety performance under high-speed collision scenarios, the collision reflex experiment was conducted as shown in Figure 10a. Operators manipulated the slave manipulator (Dobot Nova2) via the master device (Dobot Xtrainer) at velocities of 2.5 cm/s, 5 cm/s, and 10 cm/s to collide with a black hard sponge.

Four control strategies, position closed-loop velocity tracking (PL-VT), impedance control with low stiffness (Imp-L), a recent robot contact reflex (RCR) [30] and the proposed BITC method, were tested. Interaction forces and end-effector trajectories of both the master device and the slave manipulator were recorded during collisions to evaluate the proposed method’s responsiveness under extreme interaction conditions.

The Figure 10b demonstrates that the higher contact velocity resulted in a deeper interaction between the slave manipulator and the object of the test #1. The PL-VT method shows the largest penetration depth, while both the BITC and Imp-L methods exhibit compliance with relatively smaller contact depths. Moreover, when using the BITC method, the manipulator demonstrates a noticeable rebound after a collision, indicating its ability to achieve a rebound effect. Following the rebound, the end-effector of the manipulator gradually re-establishes contact with the object’s surface.

**Figure 11:**
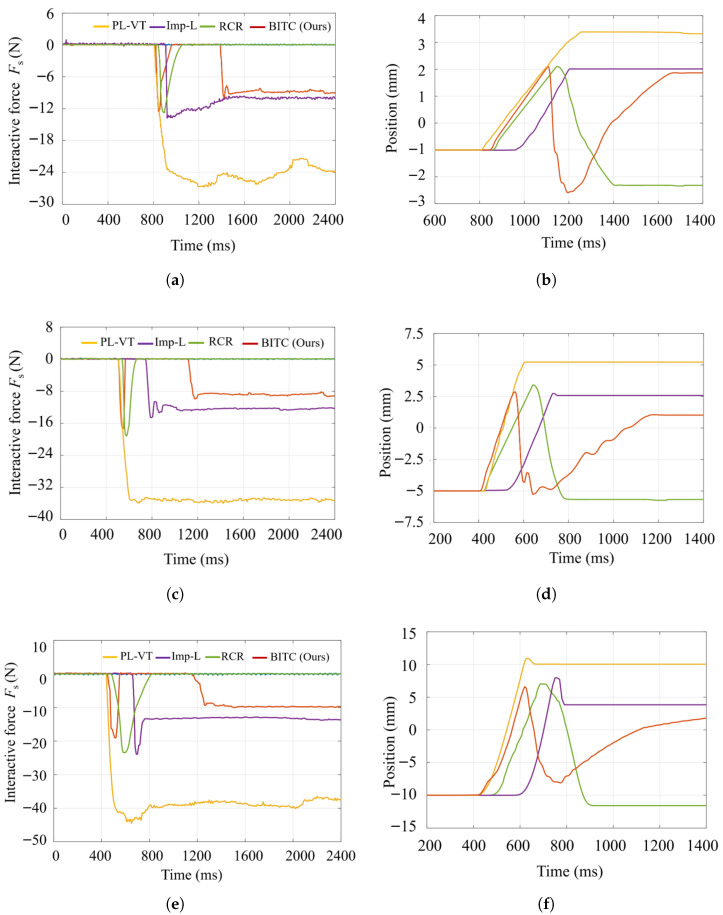
Comparison of interaction forces (**left column**) and end-effector positions (**right column**) in the collision experiment with different methods. (**a**) The contact force of test #1 (2.5 cm/s). (**b**) The end position of test #1 (2.5 cm/s). (**c**) The contact force of test #2 (5 cm/s). (**d**) The end position of test #2 (5 cm/s). (**e**) The contact force of test #3 (10 cm/s). (**f**) The end position of test #3 (10 cm/s).

The detailed results, as shown in Figure 11 (left column for force profiles, right column for trajectories), demonstrate significant differences in the collision behavior of the three methods and different collision situations. Rapid collisions induce sharp force spikes which significantly exceed those observed in the slow contact experiment. The PL-VT method, characterized by high stiffness, generates the largest interaction forces. Due to the slave manipulator’s inability to track the master’s target trajectory when obstructed, these forces persist for extended durations. In contrast, Imp-L reduces peak forces by approximately 50% through motion lag but still sustains relatively high interaction forces. The proposed method, however, exhibits a distinct safety mechanism: upon detecting abrupt force changes, the robot rapidly retracts upward, creating a sharp force spike followed by immediate rebound. This response ensures zero interaction force after rebound, with the peak force reduced by over 50% compared to PL-VT.

Notably, following the rebound, the proposed method leverages a stress-compensation delay decay mechanism, enabling the slave manipulator to slowly re-engage the object and realign with the master trajectory. During re-contact, the interaction force stabilizes at levels comparable to Imp-L and even slightly lower under higher collision speeds (e.g., 10 cm/s). This validates the method’s superior compliance over Imp-L in extreme scenarios. Two metrics (collision reaction latency and collision force attenuation) are calculated in Table 3. The collision reaction latency means the time from collision detection to the robotic arm’s reflexive withdrawal from the object surface, while the collision force attenuation is the ratio of the peak interaction force during collision to the peak force under closed-loop velocity servoing. The results in Table 3 show that our method consistently achieves shorter collision reaction latency across all three test conditions compared to RCR, likely due to the use of nonlinear reaction control gains that enable rapid responses. Specifically, in Trial 3 at a motion speed of 0.1 m/s, our method achieved a reaction time of 185 ms (within 200 ms), outperforming the recently proposed high-performance method (238 ms) by 53 ms. Notably, all results fall below the median human reaction time of 225 ms, demonstrating faster hazard avoidance than manual human operation. Regarding collision force attenuation, Imp-L, RCR [30], and BITC all reduce contact forces during collisions. RCR and BITC outperform Imp-L in Experiments 1 and 3 but show slightly lower values in Experiment 2, though the differences are minimal. Particularly in Trial 3, BITC achieves a collision force attenuation of 65.9%, with a clear trend of enhanced force suppression as velocity increases.

Quantitative analysis further highlights the proposed method’s robustness. For PL-VT and Imp-L, both dynamic peak forces (up to 22 N) and steady-state forces escalate linearly with collision velocity. In contrast, the proposed method (BITC) limits dynamic peaks to 12–18 N and steady-state forces to <17 N across all trials. Specifically, when the tracking speed is 0.1 m/s, the proposed method achieves a peak force of 18 N and a steady-state force of 16 N, outperforming Imp-L (peak: 22 N, steady-state: 18 N). These results validate the method’s robustness in ensuring stress safety across varying collision velocities, offering enhanced protection for both the manipulator and target objects.

In summary, the proposed method achieves a critical balance between force fidelity during initial interaction and compliant safety during deep penetration, outperforming traditional approaches in both robustness and adaptability. Its ability to suppress force escalation while maintaining tracking accuracy ensures safer and more reliable teleoperation in constrained environments.

## 5. Discussion

The proposed BITC framework demonstrates effective performance in maintaining interaction performance, including tracking accuracy, contact compliance, and collision reaction, for teleoperated systems in the validated scenarios. However, the scalability and robustness of this approach in more complex real-world settings, particularly for in vivo medical applications, warrant further discussion.

### 5.1. Scalability to High-DOF Manipulators and High-Latency Networks

Our control strategy is primarily deployed on the follower robot and operates in the Cartesian space at the end-effector level, utilizing velocity control. This design choice ensures compatibility with common multi-DOF manipulators (e.g., 6-axis or 7-axis robotic arms). For standard industrial or collaborative robots, the inverse kinematics solutions provided by their native controllers can be directly utilized. For custom-built systems, open-source libraries like Pinocchio [33] can be adopted for kinematic resolution. Furthermore, since the core method runs on the follower side, it possesses an inherent degree of isolation from communication latency. This is particularly significant in high-latency scenarios, where a delayed follower is more susceptible to collisions due to its inability to promptly respond to master commands. Therefore, implementing our collision reaction method in such environments is highly relevant and practical.

### 5.2. Robustness in Unstructured Environments with High Disturbances

The experimental contacts and collisions in this study were essentially unforeseen from the perspective of the follower robot, simulating a key challenge of unstructured environments. The results indicate that our method exhibits a certain level of robustness in such scenarios. The collision reflex, being a localized reactive strategy, does not rely on prior environmental perception or autonomous planning, making it applicable where sensor-based models are unavailable or unreliable. The human operator remains a crucial part of the control loop, leveraging their advanced perception and decision-making capabilities to overcome high-level disturbances through teleoperation. It is also noteworthy that the reflex distance is typically small—sufficient only to mitigate sustained contact force, which generally prevents secondary collisions caused by excessive recoil motions.

### 5.3. Limitations

A primary limitation of the current study is the lack of explicit analysis of the system’s stability under bilateral force feedback control when the collision reflex is activated on the follower side. This is critical for ensuring safe and transparent teleoperation and will be a central focus of our subsequent research.

Building upon the promising results and to directly address the scalability concerns, our future work will focus on two key enhancements:

1. Trajectory-Aware Reflex: We will develop a more sophisticated collision reaction strategy where the reflex motion is not arbitrary but is constrained to retreat along the recent trajectory of the end-effector. This will minimize unpredictable movements and further reduce the risk of secondary collisions.
2. Force-Direction Weighting: The reflex method will be extended to incorporate a weighted consideration of the contact force’s normal direction, allowing for a more intelligent and compliant withdrawal from the collision.

By integrating these advancements, we aim to significantly improve the adaptability and robustness of the BITC framework for complex in vivo teleoperation scenarios characterized by high-DOF manipulators, communication constraints, and highly uncertain environments.

## 6. Conclusions

Balancing tracking speed and interactive compliance remains a critical challenge to achieving accurate and safe teleoperation control. Conventional methods based exclusively on high-stiffness servo control or low-stiffness impedance/admittance control fail to resolve this dilemma. To address the inherent barrier, this study has proposed a bio-inspired control method (BITC) that integrates human withdrawal reflex mechanisms with shear-thickening fluid characteristics, achieving a unified balance between rapid, compliant, and safe teleoperation. The effectiveness of the control framework has been experimentally validated. BITC has emulated human stress responses to hazardous interactions by implementing a dynamic force-feedback-driven collision disengagement strategy, which reduces peak contact forces by approximately 60% compared with the high-stiffness servo control method, with comparable performance to low-stiffness impedance control. Meanwhile, the collision contact duration can be minimized to less than 120 ms. Inspired by the nonlinear stiffness properties of shear-thickening fluids, BITC establishes a nonlinear contact compliant control module with full state feedback, which achieves superior tracking performance compared to the high-stiffness method (position-closed-loop velocity tracking (PL-VT)) during free-motion phases, while maintaining steady-state force fluctuations within 3 N during sustained contact. It could enhance tracking agility and precision while maintaining compliance and safety during late-stage, slow interactions, and achieve superior force fidelity compared to impedance control. This proposed BITC method offers a comprehensive solution for teleoperation control in complex interaction environments, striking a balance between precision, compliance, and safety.

Future work will focus on extending BITC to bilateral force feedback architectures and integrating iterative learning algorithms for self-tuning controller parameters, with the aim of improving cross-task adaptability.

## Figures and Tables

**Table 1:** Maximum and average tracking errors in unconstrained teleoperation experiment. Unit: cm.

OL-VT		PL-VT		Imp-L		Imp-H		V-Imp [27]		BITC	
**Max**	**Mean**	**Max**	**Mean**	**Max**	**Mean**	**Max**	**Mean**	**Max**	**Mean**	**Max**	**Mean**
1.01	0.53	0.67	0.25	1.26	0.46	1.20	0.72	0.69	0.41	**0.48**	**0.23**
1.79	0.84	1.31	0.67	3.21	1.58	2.22	0.88	0.96	0.55	**0.94**	**0.49**
2.35	1.18	2.60	0.82	3.68	2.07	2.28	1.40	2.66	1.01	**1.52**	**0.69**
4.77	1.91	7.14	2.40	4.91	2.80	5.61	2.25	2.91	1.47	**1.91**	**1.08**

**Table 2:** The data of the slow contact experiment.

	Trial	PL-VT	Imp-L	V-Imp [27]	BITC
Max.	#1	−2.745	−0.526	−0.839	−1.673
Dynamic	#2	−5.208	−1.346	−2.723	−3.479
Force	#3	−10.26	−3.409	−7.590	−5.312
Ave.	#1	−2.371	−0.474	−0.744	−1.558
Static	#2	−4.632	−1.182	−1.927	−1.893
Force	#3	−9.748	−3.103	−2.652	−1.977

**Table 3:** The data of the collision experiment.

Collision	Trial	Imp-L	RCR [30]	BITC
Reaction	#1	-	157 ms	119 ms
	#2	-	211 ms	169 ms
Latency	#3	-	238 ms	185 ms
Collision	#1	47.4%	49.4%	58.4%
Force	#2	57.3%	52.6%	56.2%
Attenuation	#3	49.6%	56.3%	65.9%

## Data Availability

This article includes the original contributions of this research. If you have any questions, please contact the corresponding author.

## Supplementary Materials

The following supporting information can be downloaded at: https://www.mdpi.com/article/10.3390/biomimetics10090625/s1.

## Appendix A. Tuning Methodology

Our teleoperation control method is primarily divided into three parts: tracking, contact compliance, and collision reaction. The parameters for each part are shown in the Table A1. The parameter adjustment process for the three control components is as follows:

**1. Adjustment of the Tracking Control Component**

(a) First, adjust the proportional gain $k_{p}^{t}$ with the objective of minimizing the steady-state tracking error, ensuring the follower accurately tracks the leader at lower velocities while maintaining minimal overshoot.

(b) Subsequently, adjust the velocity gain $k_{v}^{t}$ to compensate for the insufficient response of the $k_{p}^{t}$ term under the overshoot constraint, thereby balancing overshoot and response performance.

(c) Finally, adjust the acceleration gain $k_{a}^{t}$. This term provides gain for acceleration feedback, which can partially mitigate tracking errors induced by communication delays, particularly during periods of significant velocity variation. However, it should be noted that acceleration measurements in robotic arms are often contaminated with substantial noise. Therefore, this parameter must be tuned cautiously, and its selected value should not be excessively large.

**Table A1:** BITC Parameter List.

Parameter Categories	Parameter Symbol	Parameter Meaning
**Tracking Control** **Parameters**	$\lambda_{t}$	Tracking control gain coefficient
	$k_{p}$	Master-slave position error-induced tracking gain coefficient
	$k_{v}$	Master-slave velocity error-induced tracking gain coefficient
	$k_{a}$	Master-slave acceleration error-induced tracking gain coefficient
**Contact Compliance** **Parameters**	$\lambda_{c}$	Contact compliance control gain coefficient
	$k_{f}^{t}$	Mapping coefficient from threshold-normalized interaction force to velocity control output
	$F_{\mathrm{th}}$	Sustained contact force threshold
	*l*	Sustained interaction force index gain
	*D*	Damping coefficient
	*n*	Non-Newtonian fluid velocity term index gain coefficient
	*K*	Stiffness coefficient
	*m*	Non-Newtonian fluid position term index gain coefficient
**Collision reaction** **parameters**	$\lambda_{r}$	Collision reaction control gain coefficient
	$C_{\mathrm{th}}$	Collision detection threshold
	$k_{e}$	Collision reaction force attenuation coefficient

**2. Adjustment of the Contact Compliance Control Component**

(a) Initially, set the sustained deep contact force threshold $F_{\mathrm{th}}$ based on safety requirements. This value is user-defined based on the desired level of interaction safety; for instance, in our experiments, it was set to 2 N.

(b) Next, adjust the index gain *l*. This parameter is critically related to the compliant transition behavior as the contact force approaches the threshold $F_{\mathrm{th}}$. A larger *l* value results in a more responsive compliant transition near the force threshold, causing the impedance to appear smaller. Conversely, a smaller *l* value leads to a more sluggish response, blurring the distinction between safe contact and potentially hazardous deep contact.

(c) Then, adjust the force gain $k_{f}^{t}$. This gain is primarily intended to scale the control input derived from the contact force into the velocity control output range of the robotic arm system. Since ${\left(F_{s}/F_{\mathrm{th}}\right)}^{l}$ is a dimensionless quantity, $k_{f}^{t}$ must be adjusted to match the command range of the velocity control.

(d) Proceed to adjust the damping coefficient *D*, the non-Newtonian fluid velocity term exponential gain coefficient *n*, the stiffness coefficient $k_{e}$, and the non-Newtonian fluid position term exponential gain coefficient *m*. The damping coefficient *D* suppresses the response during the compliant transition. When the compliant reaction force gain $k_{f}^{t}{\left(F_{s}/F_{\mathrm{th}}\right)}^{l}$ is relatively large, increasing *D* can prevent the robotic arm from losing contact with the surface during the transition to compliant interaction. The non-Newtonian fluid position and velocity term exponential gain coefficients *m* and *n* are associated with the responsiveness of reactive motions during collision. Based on the characteristics of shear-thickening fluids, *m* and *n* typically lie between 0 and 1. This formulation simulates an effect where faster movement results in decreased damping and elasticity, facilitating rapid withdrawal from contact upon collision reaction. Simultaneously, it preserves the energy dissipation capability, which contributes to the overall stability of the system. Finally, adjust the stiffness coefficient *K*. A larger *K* generally yields higher fidelity to position commands, whereas a smaller *K* enhances compliance.

**3. Adjustment of the Collision Reaction Control Component**

(a) Adjust the collision threshold $C_{\mathrm{th}}$ such that it is not triggered during rapid reciprocating motion but is reliably triggered upon the occurrence of an actual collision.

(b) Initially, set the attenuation coefficient $k_{e}$ to a moderate predetermined value, for example, $k_{e}=1$ (the calculation result is as shown previously in the manuscript). Adjust the gain $\lambda_{c}$ to achieve the desired effect where the robotic arm generates an immediate retreat response upon collision detection.

(c) Subsequently, adjust the attenuation coefficient $k_{e}$ according to the required task recovery time. A larger $k_{e}$ causes the control output generated by the collision reaction to decay more rapidly, leading to faster task recovery. However, an excessively large $k_{e}$ may result in an overly short duration of the collision reaction, preventing its effect from being fully realized.

**4. Integrated Control System Tuning**

Comprehensively adjust the control parameter $\lambda_{t}$, $\lambda_{c}$, $\lambda_{r}$ (or the blending parameters/weights) across the three modules to achieve a balanced or optimal overall performance, ensuring that the objectives of each module are adequately met without undue compromise.

## References

[1] Jiang Z., Ma Y., Cao X., Shen M., Yin C., Liu H., Cui J., Sun Z., Huang X., Li H. (2023). FC-EODR: Immersive Humanoid Dual-Arm Dexterous Explosive Ordnance Disposal Robot. Biomimetics.

[2] Cheng C., Dai W., Wu T., Chen X., Wu M., Yu J., Jiang J., Lu H. (2024). Efficient and Precise Homo-Hetero Teleoperation Based on an Optimized Upper Limb Exoskeleton. IEEE/Asme Trans. Mechatronics.

[3] Saafi H., Laribi M.A., Zeghloul S. (2020). Forward Kinematic Model Resolution of a Special Spherical Parallel Manipulator: Comparison and Real-Time Validation. Robotics.

[4] Artigas J., Balachandran R., Riecke C., Stelzer M., Weber B., Ryu J.H., Albu-Schaeffer A. KONTUR-2: Force-feedback teleoperation from the international space station. Proceedings of the 2016 IEEE International Conference on Robotics and Automation (ICRA).

[5] Pei X., Liu S., Wei A., Shi R., Dai Z. (2023). Bioinspired Rigid–Flexible Coupled Adaptive Compliant Motion Control of Robot Gecko for Space Stations. Biomimetics.

[6] Hogan N. Impedance Control: An Approach to Manipulation. Proceedings of the American Control Conference.

[7] Colgate E., Hogan N. An analysis of contact instability in terms of passive physical equivalents. Proceedings of the International Conference on Robotics and Automation.

[8] Chang Y.H., Yang C.Y., Lin H.W. (2024). Robust Adaptive-Sliding-Mode Control for Teleoperation Systems with Time-Varying Delays and Uncertainties. Robotics.

[9] Zhai D.H., Xia Y. (2017). Adaptive Control of Semi-Autonomous Teleoperation System with Asymmetric Time-Varying Delays and Input Uncertainties. IEEE Trans. Cybern..

[10] Vorndamme J., Melone A., Kirschner R., Figueredo L., Haddadin S. (2025). Safe Robot Reflexes: A Taxonomy-Based Decision and Modulation Framework. IEEE Trans. Robot..

[11] Yang C., Ganesh G., Haddadin S., Parusel S., Albu-Schaeffer A., Burdet E. (2011). Human-Like Adaptation of Force and Impedance in Stable and Unstable Interactions. IEEE Trans. Robot..

[12] Haddadin S., De Luca A., Albu-Schäffer A. (2017). Robot Collisions: A Survey on Detection, Isolation, and Identification. IEEE Trans. Robot..

[13] Tomić T., Ott C., Haddadin S. (2017). External Wrench Estimation, Collision Detection, and Reflex Reaction for Flying Robots. IEEE Trans. Robot..

[14] Phan-Thien N., Dudek J. (1982). Pulsating flow of a plastic fluid. Nature.

[15] Brown E., Forman N.A., Orellana C.S., Zhang H., Maynor B.W., Betts D.E., DeSimone J.M., Jaeger H.M. (2010). Generality of shear thickening in dense suspensions. Nat. Mater..

[16] Keemink A.Q., Van der Kooij H., Stienen A.H. (2018). Admittance control for physical human–robot interaction. Int. J. Robot. Res..

[17] Ferraguti F., Landi C.T., Sabattini L., Bonfe M., Fantuzzi C., Secchi C. (2019). A variable admittance control strategy for stable physical human–robot interaction. Int. J. Robot. Res..

[18] Lu Z., Huang P., Liu Z., Chen H. (2019). Fuzzy-Observer-Based Hybrid Force/Position Control Design for a Multiple-Sampling-Rate Bimanual Teleoperation System. IEEE Trans. Fuzzy Syst..

[19] Du L., Lv J., Chen S., Qiu L. Adaptive Impedance Modulation-Based Control for Human-Robot Interaction. Proceedings of the IEEE International Conference on Robotics, Automation and Mechatronics (RAM).

[20] Xia W., Liao Z., Lu Z., Yao L. (2025). Bio-Signal-Guided Robot Adaptive Stiffness Learning via Human-Teleoperated Demonstrations. Biomimetics.

[21] Tee K.P., Burdet E., Chew C.M., Milner T.E. (2004). A model of force and impedance in human arm movements. Biol. Cybern..

[22] Li Y., Jarrassé N., Burdet E., Laumond J.P., Mansard N., Lasserre J.B. (2017). Versatile Interaction Control and Haptic Identification in Humans and Robots. Geometric and Numerical Foundations of Movements.

[23] Jin Z., Qin D., Liu A., Zhang W.a., Yu L. (2023). Model Predictive Variable Impedance Control of Manipulators for Adaptive Precision-Compliance Tradeoff. IEEE/ASME Trans. Mechatronics.

[24] Michel Y., Abdelhalem Y., Cheng G. (2024). Passivity-Based Teleoperation with Variable Rotational Impedance Control. IEEE Robot. Autom. Lett..

[25] Yang Y., Constantinescu D., Shi Y. (2020). Robust Four-Channel Teleoperation Through Hybrid Damping-Stiffness Adjustment. IEEE Trans. Control Syst. Technol..

[26] Michel Y., Li Z., Lee D. (2023). A Learning-Based Shared Control Approach for Contact Tasks. IEEE Robot. Autom. Lett..

[27] Michel Y., Rahal R., Pacchierotti C., Giordano P.R., Lee D. (2021). Bilateral Teleoperation with Adaptive Impedance Control for Contact Tasks. IEEE Robot. Autom. Lett..

[28] Harder M., Iskandar M., Lee J., Dietrich A. Extensions to Dynamically-Consistent Collision Reaction Control for Collaborative Robots. Proceedings of the 2023 IEEE/RSJ International Conference on Intelligent Robots and Systems (IROS).

[29] Gao Z., Peng F., Chen C., Zhang Y., Wang Y., Yan R., Tang X. (2024). Event-triggered Hybrid Force Feedback Architecture with Tank-based Stabilization Method for Complicated Bilateral Teleoperation Tasks. Int. J. Control. Autom. Syst..

[30] Vorndamme J., Figueredo L., Haddadin S. Robot Contact Reflexes: Adaptive Maneuvers in the Contact Reflex Space. Proceedings of the 2022 IEEE/RSJ International Conference on Intelligent Robots and Systems (IROS).

[31] Cao Y., Ma S., Zhang M., Li Z., Liu J., Huang J., Zhang Z.Q. (2025). Musculoskeletal Model-Based Adaptive Variable Impedance Control With Flexible Prescribed Performance for Rehabilitation Robots. Ieee/Asme Trans. Mechatronics.

[32] Chen L., Chen L., Chen X., Lu H., Zheng Y., Wu J., Wang Y., Zhang Z., Xiong R. (2024). Compliance while resisting: A shear-thickening fluid controller for physical human-robot interaction. Int. J. Robot. Res..

[33] Carpentier J., Saurel G., Buondonno G., Mirabel J., Lamiraux F., Stasse O., Mansard N. The Pinocchio C++ library—A fast and flexible implementation of rigid body dynamics algorithms and their analytical derivatives. Proceedings of the IEEE International Symposium on System Integrations (SII).

